# Renal Tubular Epithelial CRLF1 Interacts With ITGB1 to Accelerate Fibrosis During the Transition From AKI to CKD

**DOI:** 10.1002/advs.76896

**Published:** 2026-07-30

**Authors:** Chunjie Wang, Yan Zhang, Fang Bai, Shankui Qian, Feng Feng, Fangyi Lu, Jiahui Fan, Kuipeng Yu, Xiangdong Yang

**Affiliations:** ^1^ Department of Nephrology Qilu Hospital of Shandong University Jinan Shandong China; ^2^ Laboratory of Basic Medical Sciences Qilu Hospital of Shandong University Jinan Shandong China; ^3^ Department of Neurosurgery Qilu Hospital Cheeloo College of Medicine and Institute of Brain and Brain‐Inspired Science Shandong University Jinan China

**Keywords:** AKI‐to‐CKD transition, CRLF1, fibrosis, ITGB1, SMAD3

## Abstract

Acute kidney injury (AKI) frequently progresses to chronic kidney disease (CKD), ultimately leading to end‐stage renal failure. Cytokine receptor‐like factor 1 (CRLF1) is a secreted protein with low or undetectable expression in normal physiology, but it is transcriptionally and translationally upregulated under pathological conditions. In this study, we found CRLF1 to be upregulated in the kidneys of patients with ATN and CKD, positively correlated with renal interstitial fibrosis, and negatively correlated with renal function. In vivo functional inhibition studies demonstrated that CRLF1 suppression significantly improved renal function, mitigated pathological damage, and delayed fibrosis progression in mouse models of IRI or UUO. Further in vitro studies revealed that CRLF1 promotes fibrosis and inflammation in renal tubular epithelial cells (TECs). Mechanistically, we employed chromatin immunoprecipitation (ChIP) to validate SMAD3 binding to the CRLF1 promoter and its transcriptional regulation, while elevated CRLF1 interacts with the VWFA domain of integrin β1 (ITGB1). Furthermore, transcriptomic sequencing revealed that CRLF1 activates the PI3K/AKT signaling pathway, promoting tubular epithelial cell fibrosis progression via ITGB1. These findings underscore the critical role of CRLF1 in renal fibrosis progression and suggest its potential as a therapeutic target for inhibiting the progression from AKI to CKD.

## Graphical abstract

During renal fibrosis, SMAD3 acts as a transcription factor for CRLF1, promoting its expression and secretion. CRLF1 then binds to ITGB1 via an autocrine mechanism, activating the PI3K‐AKT signaling pathway to mediate renal fibrosis. This accelerates the progression from AKI to CKD, highlighting the therapeutic potential of targeting CRLF1 for alleviating renal fibrosis.

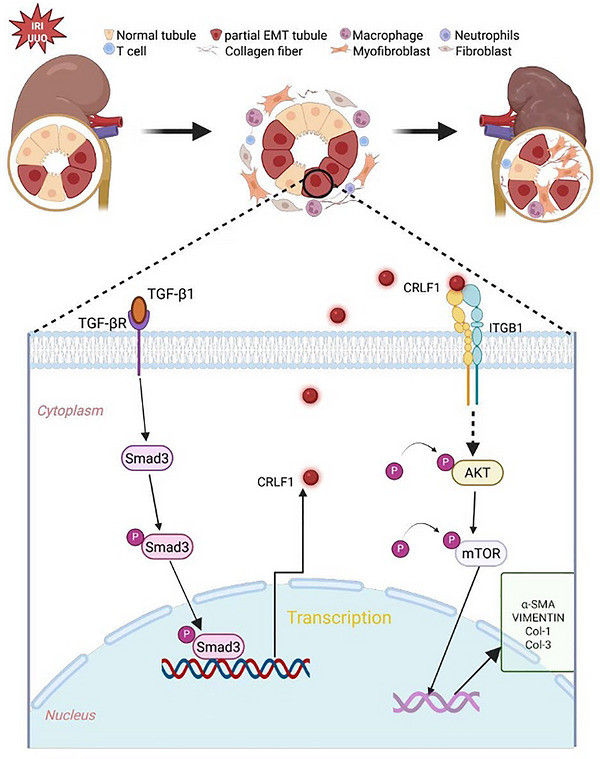

## 1 Introduction

The global prevalence of chronic kidney disease (CKD) among adults is approximately 10%, and its incidence, prevalence, and mortality have increased significantly worldwide [1, 2]. Acute kidney injury (AKI) is characterized by high incidence and mortality rates, rendering it a major concern in the global public health arena. Renal fibrosis is characterized by the gradual accumulation of extracellular matrix components within renal tissues. Notably, persistent or extensive fibrosis in the context of AKI contributes to the progression of CKD and end‐stage renal failure (ESRD) [3, 4]. Renal tubular epithelial cells (TECs) play an indispensable role in this process [5, 6]. Impaired TECs drive the pathogenesis of tubulointerstitial fibrosis and the progression of chronic kidney disease via the secretion of fibrogenic mediators, such as transforming growth factor‐β (TGF‐β), connective tissue growth factor (CTGF), and NOD‐like receptor pyrin domain‐containing 3 (NLRP3) [7, 8, 9, 10, 11]. The exact mechanism driving the progression of renal fibrosis has not yet been fully elucidated [12]. The search for new therapeutic targets against this process could represent an important approach to improving disease outcomes.

Cytokine receptor‐like factor 1 (CRLF1) is a secreted protein and belongs to the cytokine receptor‐like factor family. CRLF1 is highly conserved among vertebrates, with 96% amino acid sequence similarity of CRLF1 between mice and humans reaching 96% [13]. Currently, CRLF1 has been recognized as a clinical auxiliary diagnostic marker for cold‐induced sweating syndrome (CISS) [14, 15]. Previous studies have proposed that CRLF1 is typically secreted as a heterodimer by binding to the cytokine CLCF1 [16]. However, recent research has revealed that CRLF1 may exert independent biological functions [17]. Studies have demonstrated that CRLF1 can upregulate the expression of collagen type III (Col‐III) and the expression of α‐smooth muscle actin (α‐SMA) in quiescent hepatic stellate cells (HSCs) [18]. CRLF1 exacerbates cardiac fibrosis by activating the ERK1/2 signaling pathway [19]. Inhibiting CRLF1 promotes chondrogenic differentiation of BMSCs and protects cartilage tissue from osteoarthritis damage by activating miR‐320 [20]. CRLF1 knockdown reduces fibrosis induced by inflammation and mechanical stress [21]. However, the role of CRLF1 in kidney injury remains unexplored.

This study confirmed that the mRNA and protein levels of CRLF1 remained elevated in both human patients and a mouse model of AKI‐to‐CKD transition. Moreover, its expression was positively correlated with the severity of chronic kidney disease (CKD) and renal fibrosis. Knockdown of CRLF1 inhibited TGF‐β1‐induced cellular fibrosis. By analyzing RNA sequencing data from the renal tubular epithelial cell (TEC) fibrosis model combined with mass spectrometry (MS) analysis, we identified an association between CRLF1 and ITGB1. Further co‐immunoprecipitation (Co‐IP) experiments demonstrated that CRLF1 binds to the VWFA domain of integrin β1. In addition, overexpression of ITGB1 abrogated the renal protective effect of CRLF1 gene knockdown in mice with IRI or UUO. Collectively, these findings indicate that CRLF1 exerts a novel function in regulating renal fibrosis and provide a potential therapeutic strategy for AKI to CKD.

## 2 Materials and Methods

### 2.1 Human Kidney Tissue Samples

Human kidney biopsy specimens were obtained from patients diagnosed with acute tubular necrosis (ATN) and chronic kidney disease (CKD), as well as from individuals who underwent nephrectomy for tumors and had no evidence of ATN, CKD, or other renal diseases. All specimens were collected from the Department of Pathology at Qilu Hospital of Shandong University. This study was approved by the Shandong University Research Ethics Committee (Approval No: KYLL‐2020(KS)‐030), and informed consent was obtained from all patients.

### 2.2 Animal Models

C57BL/6J mice were obtained from the Experimental Animal Center of Shandong University (Jinan, China). All the protocols implemented in this study strictly adhered to the regulatory guidelines sanctioned by the Animal Care and Use Committee of Shandong University.

IRI and UUO models were induced in 6–8‐week‐old C57BL/6J mice. Specifically, the experimental mice were anesthetized with approximately 50 mg/kg sodium pentobarbital, and their body temperature was maintained at 37°C. Bilateral renal pedicles were clamped with microvascular clamps for 30 min. Each sham‐operated mouse underwent identical surgical procedures except for the absence of vascular clamping. Blood and kidney samples were collected from mice at 2, 7, and 14 days after IRI [22, 23, 24]. For the UUO‐induced renal fibrosis model, the unilateral ureter was ligated, and kidney tissues were harvested at 3, 7, and 14 days [25, 26].

To downregulate CRLF1 expression, we followed the previous approach. Kidneys were exposed and injected with lv‐CRLF1 or lv‐NC (2×10^6^ IU per kidney; diluted in 100 µl of sterile 0.9% NaCl) at three or four locations using an insulin syringe (Table S1) [27, 28]. Two weeks later, the corresponding animal models were established.

### 2.3 Cell Culture and Treatment

Human renal tubular HK‐2 cells (National Collection of Authenticated Cell Cultures; CRL‐2190) were cultured in RPMI 1640 medium supplemented with 10% fetal bovine serum (Gibco, cat. no. 10099–141), 100 U/ml penicillin, and 0.1 mg/mL streptomycin. When reaching approximately 70% confluence, cells were serum‐starved overnight, and then treated with varying concentrations of cisplatin for different durations. LY294002 (HY‐10108, MCE), a selective PI3K inhibitor targeting PI3K to block downstream signaling activation, was used to treat HK‐2 cells at 20 µM for 24 h. SC79 (HY‐18749, MCE), a specific AKT activator targeting AKT to promote its phosphorylation activation, was used to treat HK‐2 cells at 10 µM for 2 h.

### 2.4 Histology and Immunohistochemistry (IHC)

Formalin‐fixed paraffin‐embedded sections with a thickness of 3 µm were used for immunohistochemical staining. After deparaffinization, the sections were blocked with goat serum and incubated overnight with the appropriate primary antibodies at 4°C, and then incubated with biotinylated secondary antibodies. Immunoreactive signals were developed using a chromogenic substrate. The antibodies are provided in Table S2.

### 2.5 Transfection for siRNA

For siRNA transfection, cells were plated one day prior to the transfection procedure. Transfection was carried out with Lipofectamine 3000 (YEASEN, Shanghai, China) according to the manufacturer's protocols when the cells reached approximately 70% confluency. siRNAs was purchased from Genepharma (Table S1).

### 2.6 Mass Spectrometry (MS)

HK‐2 cell lysates were immunoprecipitated with anti‐CRLF1 and protein A/G magnetic beads. The specific operating procedures are provided in the kit (KTD104, Abbkine, China). Mass Spectrometry detection and analysis were conducted by Genepharma.

### 2.7 Co‐Immunoprecipitation (Co‐IP)

Cells were lysed in lysis buffer. Subsequently, the cell lysate was incubated with the specified antibodies and the corresponding Dynabeads Protein G at 4°C overnight (KTD104, Abbkine, China). Following the incubation, Western blotting was performed to detect the target proteins. The antibodies are provided in Table S2.

### 2.8 RNA‐seq Analysis

HK‐2 cells were transfected with Si‐CRLF1 and NC and then stimulated with TGF‐β1. Total RNA was extracted from the Si ‐CRLF1 and NC groups. Subsequently, mRNA sequencing was performed by LC Bio Corporation in Hangzhou, China.

### 2.9 ChIP Analysis

ChIP assays were conducted using the SimpleChIP Plus Enzymatic Chromatin IP Kit (#9005, Cell Signaling Technology, Boston, USA). HK‐2 cells were cross‐linked with 1% formaldehyde. The cross‐linked cell lysates were sonicated to shear chromatin, followed by immunoprecipitation. ChIP signals were quantified by qRT‐PCR. The sequences utilized for ChIP are provided in Table S3.

### 2.10 Luciferase Reporter Assay

HEK293T cells were collected 48 h after transfection with the plasmid. Relative luciferase activity was detected using a dual luciferase reporter assay kit (YEASEN, Shanghai, China). Firefly luciferase activity was normalized to Renilla luciferase activity to reflect the relative reporter activity.

### 2.11 Cellular Immunofluorescence

HK‐2 cells were seeded onto coverslips and allowed to grow to confluence before being fixed with 4% paraformaldehyde. The cells were incubated with primary antibodies overnight after permeabilization and blocking. Fluorescent secondary antibody incubation was conducted the following day. DAPI and anti‐fluorescence quencher were added, and the coverslips were mounted for observation under a fluorescence microscope. The antibodies are provided in Table S2.

### 2.12 Quantitative Real‐Time Polymerase Chain Reaction (qRT‐PCR)

qRT‐PCR was performed in accordance with the protocols described previously [28, 29]. Detailed information on primers used in these experiments is provided in Table S3.

### 2.13 Western Blotting

Frozen kidney samples were homogenized with radioimmunoprecipitation assay buffer and subjected to Western blotting. The antibodies used are listed in Table S2.

### 2.14 Histological Analysis

Renal tissues were fixed in 4% paraformaldehyde for 24 h and then embedded in paraffin. Paraffin sections (3 µm thick) were subjected to deparaffinization and rehydration. Renal sections were stained with standard hematoxylin and eosin (H&E).

### 2.15 Sirius Red Staining

Mouse kidney tissue sections were dewaxed and rehydrated, then stained at room temperature for 10–15 min with Sirius Red picric acid solution. Sections were differentiated for several seconds with picric acid or glacial acetic acid. Sections were subsequently dehydrated and cleared using a graded series of solutions, mounted with neutral balsam, dried, and observed under an optical microscope.

### 2.16 Masson Staining

Renal tissues were fixed with 4% paraformaldehyde for 24 h, and then embedded in paraffin and sectioned at a thickness of 3 µm for Masson staining. Images were acquired using NIS Elements software and a Nikon Microscope Imaging System.

### 2.17 Serum Creatinine and Urea Nitrogen Assessment

Serum creatinine (Scr) and blood urea nitrogen (BUN) levels were measured using an automated biochemical analyzer (AU480, Beckman Coulter, Atlanta, USA).

### 2.18 Cellular DNA Flow Cytometric Analysis

For cell cycle detection, HK‐2 cells were seeded in 6‐well plates at a density of 2 × 10^5^ cells per well. Upon completion of the indicated treatments, cells were collected and fixed in pre‐chilled 70% ethanol at 4°C overnight. After fixation, cells were stained with propidium iodide (BD Biosciences, Cat. No. 550825) for 15 min at room temperature. Finally, cell cycle distribution was detected by flow cytometry.

### 2.19 Statistical Analyses

Statistical analyses were performed using GraphPad Prism 8.0.1 software (GraphPad Software, Inc., San Diego, CA, USA). A *t*‐test was adopted to compare two groups; for three or more groups, one‐way ANOVA was performed first, and Tukey's post‐test was then used. Statistical significance was defined with p < 0.05, and the corresponding significance levels were indicated as ^*^p < 0.05, ^**^p < 0.01, and ^***^p < 0.001.

## 3 Results

### 3.1 CRLF1 Expression Associates With Fibrosis Progression in Human and Murine Kidneys

**FIGURE 1:**
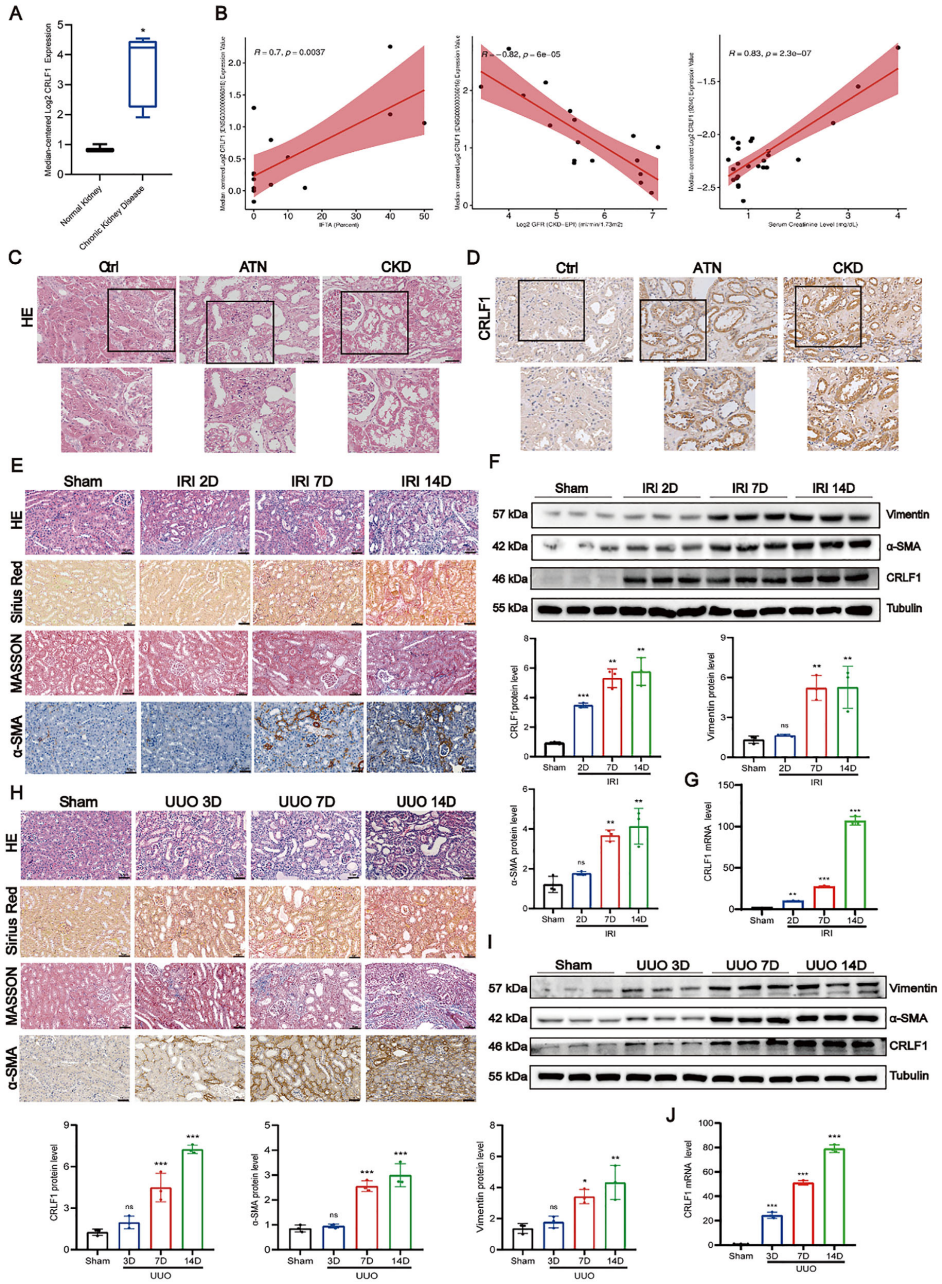
CRLF1 expression is upregulated in tubular epithelial cells (TECs) of patients with ATN and CKD and mice with IRI and UUO. (A) Analysis of renal biopsies from control (Ctrl, *n* = 3 patients) and AKI (*n* = 5 patients) cohorts. (B) Correlation analysis between CRLF1 expression and interstitial fibrosis and tubular atrophy (*n* = 14 patients), eGFR (*n* = 17 patients) and Scr (*n* = 25 patients) of Focal Segmental Glomerulosclerosis (FSGS) samples. (C) Representative images of HE staining of patients with ATN and CKD. (D) Representative images of IHC staining for CRLF1 expression in kidney tissues from patients with ATN and CKD. (E) Representative images of HE, Sirius Red, Masson and IHC for α‐SMA staining of kidney tissues on days 0, 2, 7, and 14 in IRI induced AKI‐CKD transition mouse model. (F) Representative Western blotting images and quantification of CRLF1 in IRI induced AKI‐to‐CKD transition mouse model. (*n* = 3). (G) qRT‐PCR showed the mRNA expression levels of *Crlf1* in IRI‐induced AKI‐to‐CKD transition mouse model. (*n* = 3). (H) Representative images of HE, Sirius Red, Masson and IHC for α‐SMA staining of kidney tissues on days 0, 3, 7, and 14 in UUO induced mouse model. (I) Representative Western blotting images and quantification of CRLF1 in UUO induced mouse model. (*n* = 3). (J) qRT‐PCR showed the mRNA expression levels of *Crlf1* in UUO induced mouse model. (*n* = 3). Data are presented as the mean ± SD. ^*^p < 0.05, ^**^p < 0.01, ^***^p < 0.001.

By searching bioinformatics‐related websites (https://nephroseq.org/resource/main.html), we observed a significant elevation in CRLF1 expression (Figure 1A). Further analysis revealed that the expression of CRLF1 showed a correlation with serum creatinine, interstitial fibrosis, renal tubular atrophy, and eGFR (Figure 1B). To evaluate the expression patterns of CRLF1, we enrolled biopsy samples from AKI, CKD patients and adjacent noncancerous kidney tissue from patients with renal carcinoma were performed H&E and immunohistochemical (IHC) staining (Figure 1C,D). CRLF1 expression levels were markedly elevated in the kidneys of patients with AKI and CKD compared to controls. In the mouse IRI model, Scr and BUN peaked at day 2 post‐reperfusion, reflecting severe acute renal injury. While both markers declined at days 7 and 14, they remained significantly elevated relative to the sham group, indicating sustained renal dysfunction during the progression to chronic kidney disease and validating the model (Figure S1A,B).

Severe tubular dilatation, atrophy, and inflammatory cell infiltration were observed in mouse kidneys on day 2 after IRI as previously revealed by HE staining. Masson and Sirius Red staining revealed that renal interstitial collagen deposition was significantly increased at day 7 after IRI in mice and became more pronounced at day 14 post‐surgery [30, 31] (Figure 1E). WB and qRT‐PCR confirmed that the expression of CRLF1 was significantly increased in the acute phase of IRI (Figure 1F,G and Figure S2A). Furthermore, the expression of CRLF1 increased over time in murine models of UUO (Figure 1I,J). Masson and Sirius Red staining revealed that renal interstitial collagen fibers began to increase at day 3 after UUO, with a significant increase in collagen density at day 7. By day 14, collagen deposition was extensive and dense, indicating that interstitial fibrosis progressively worsened over time following UUO (Figure 1H). qRT‐PCR further confirmed this result (Figure S2C). Immunofluorescence colocalization staining of the proximal tubule marker AQP1 and CRLF1 revealed that CRLF1 is partially expressed in the proximal tubules (Figure S2B,D).

### 3.2 Knockdown of CRLF1 Alleviates Renal IRI‐Induced Kidney Injury

**FIGURE 2:**
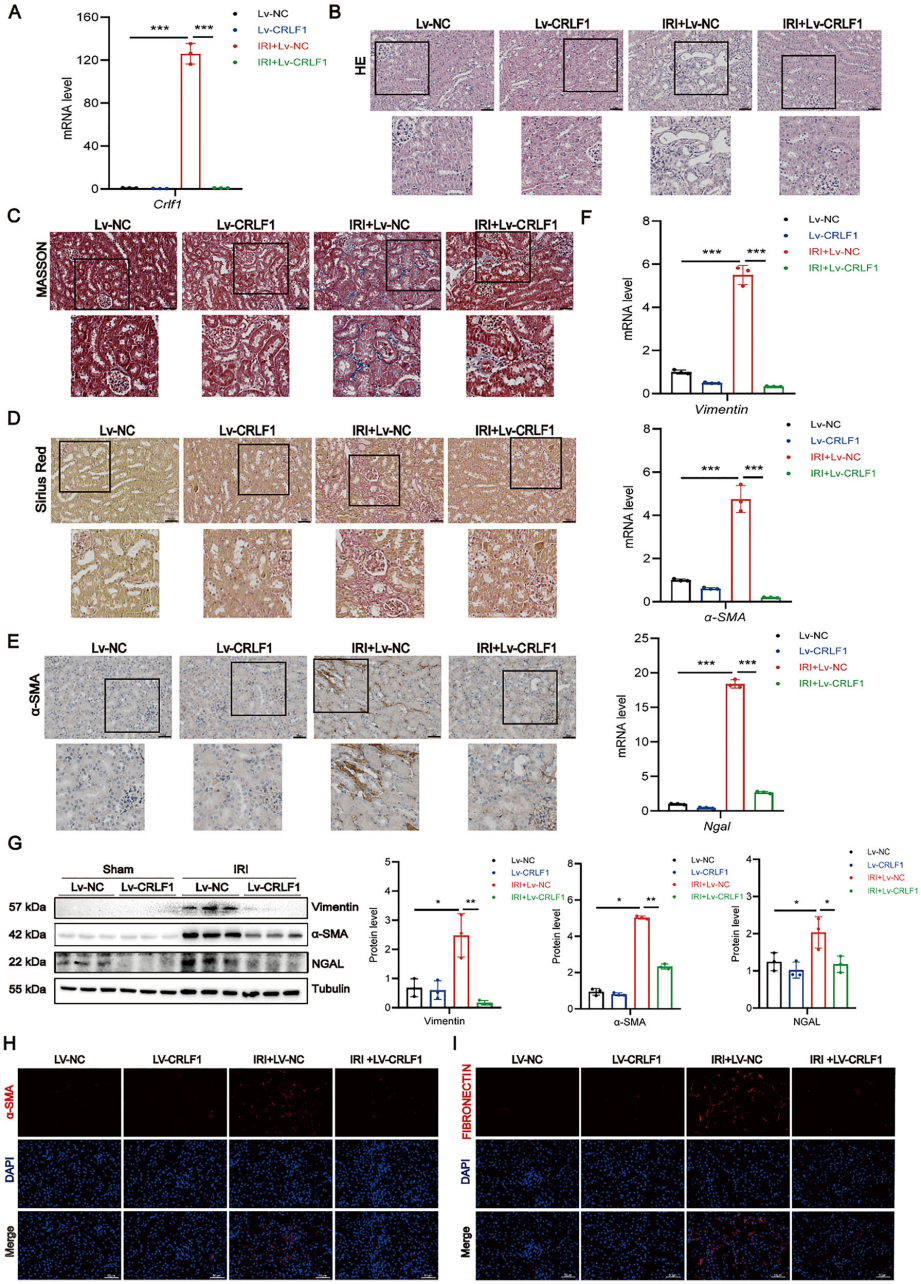
CRLF1 inhibition alleviates renal impairment and suppresses renal fibrosis in IRI‐induced AKI‐to‐CKD transition model. (A) qRT‐PCR showed the mRNA expression levels of *Crlf1* from healthy mice and mice subjected to IRI with or without lv‐NC or lv‐CRLF1. (B) Representative images of HE staining of kidney tissues from healthy mice and mice subjected to IRI with or without lv‐NC or lv‐CRLF1. Scale bar =  50 µm. (C) Representative images of Masson staining of kidney tissues from healthy mice and mice subjected to IRI with or without lv‐NC or lv‐CRLF1. Scale bar =  50 µm. (D) Representative images of Sirius Red staining of kidney tissues from healthy mice and mice subjected to IRI with or without lv‐NC or lv‐CRLF1. Scale bar =  50 µm. (E) Representative images of IHC for α‐SMA expression levels as indicated. Scale bar =  50 µm. (F) qRT‐PCR showed the mRNA expression levels of *Ngal*, *α‐SMA* and *Vimentin* of healthy mice and mice subjected to IRI with or without lv‐NC or lv‐CRLF1. (*n* = 3). (G) Representative Western blotting images and quantification of CRLF1 of healthy mice and mice subjected to IRI with or without lv‐NC or lv‐CRLF1. (*n* = 3). (H) Representative images of IF for α‐SMA expression levels as indicated. Scale bar =  50 µm. (I) Representative images of IF for fibronectin expression levels as indicated. Scale bar =  50 µm. Data are presented as the mean ± SD. ^*^p < 0.05, ^**^p < 0.01, ^***^p < 0.001.

To evaluate the role of CRLF1 in renal fibrosis in vivo, we generated CRLF1 knockdown mice. The knockdown efficiency of CRLF1 in the kidney was verified (Figure 2A and Figure S3A). In the IRI mouse model, HE, Scr and BUN assays revealed that CRLF1 knockdown significantly mitigated renal injury (Figure 2B and Figure S3B,C). Masson staining and Sirius Red staining further demonstrated reduced interstitial fibrosis in CRLF1‐knockdown mice compared with the control group (Figure 2C,D). IHC staining suggested a reduction of a fibrotic marker (α‐SMA) in CRLF1‐knockdown mice (Figure 2E). qRT‐PCR analysis of fibrotic markers, such as *Vimentin* and *α‐SMA*, suggested their decreased expression in CRLF1‐knockdown mice (Figure 2F). Western blotting analysis of NGAL, Vimentin and α‐SMA also showed quantitative evidence of their decreased expression in CRLF1‐knockdown mice, underscoring its potential involvement in renal fibrosis (Figure 2G). Immunofluorescence staining of fibrotic indicators fibronectin and α‐SMA also showed that their expression levels were significantly reduced in CRLF1‐knockdown mice (Figure 2H,I). These results suggested that CRLF1 inhibition mitigates renal pathological damage in IRI mice and delays the progression of renal fibrosis.

### 3.3 Knockdown of CRLF1 Attenuates UUO‐Induced Renal Fibrosis

**FIGURE 3:**
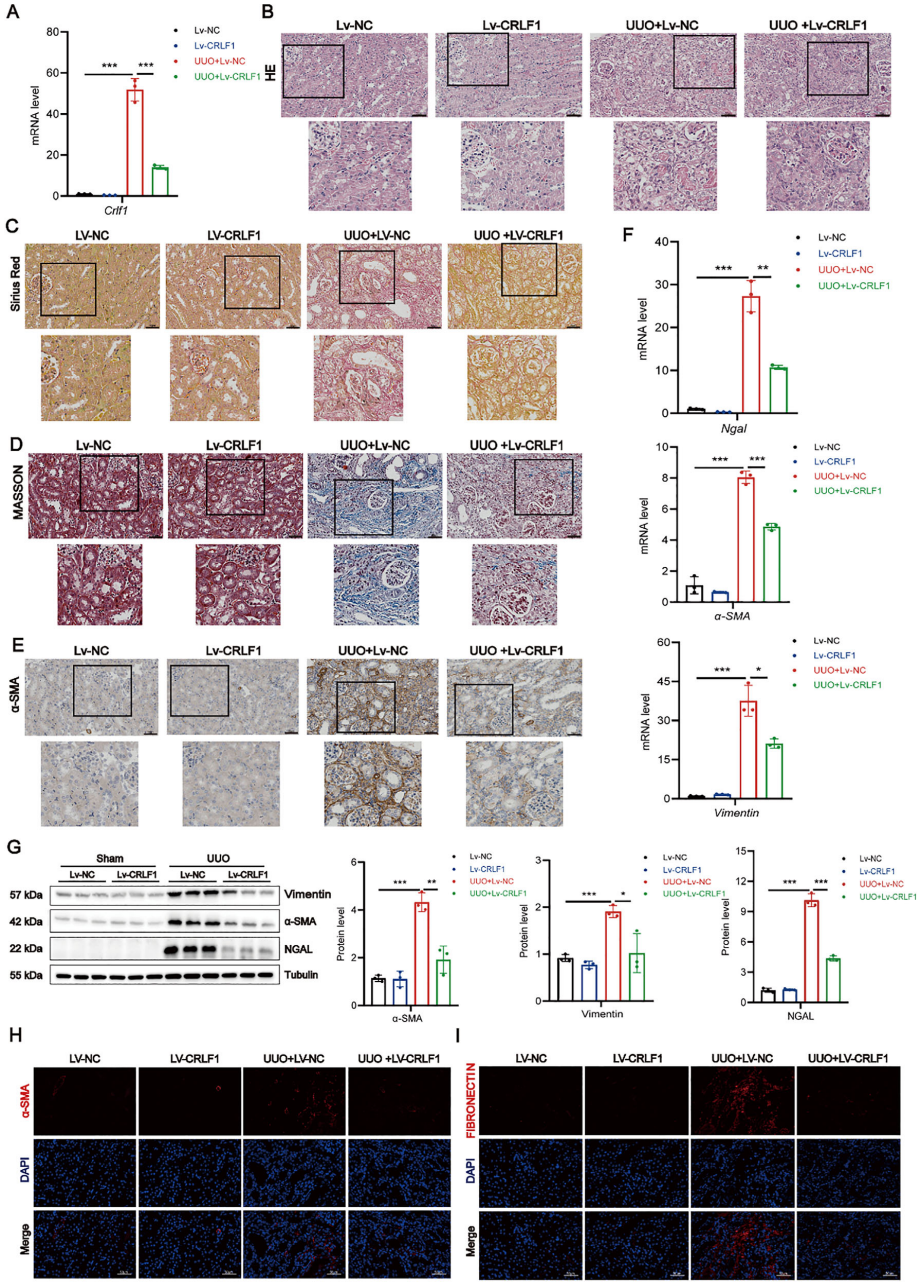
CRLF1 inhibition protects against kidney dysfunction and delays renal fibrosis in UUO‐induced mouse model. (A) qRT‐PCR showed the mRNA expression levels of *Crlf1* from healthy mice and mice subjected to UUO with or without lv‐NC or lv‐CRLF1. (B) Representative images of HE staining of kidney tissues from healthy mice and mice subjected to UUO with or without lv‐NC or lv‐CRLF1. Scale bar =  50 µm. (C) Representative images of Sirius Red staining of kidney tissues from healthy mice and mice subjected to UUO with or without lv‐NC or lv‐CRLF1. Scale bar =  50 µm. (D) Representative images of Masson staining of kidney tissues from healthy mice and mice subjected to UUO with or without lv‐NC or lv‐CRLF1. Scale bar =  50 µm. (E) Representative images of IHC for α‐SMA expression levels as indicated. Scale bar =  50 µm. (F) qRT‐PCR showed the mRNA expression levels of *Ngal*, *α‐SMA* and *Vimentin* of healthy mice and mice subjected to UUO with or without lv‐NC or lv‐CRLF1. (*n* = 3). (G) Representative Western blotting images and quantification of CRLF1 of healthy mice and mice subjected to UUO with or without lv‐NC or lv‐CRLF1. (*n* = 3). (H) Representative images of IF for α‐SMA expression levels as indicated. Scale bar =  50 µm. (I) Representative images of IF for fibronectin expression levels as indicated. Scale bar =  50 µm. Data are presented as the mean ± SD. ^*^p < 0.05, ^**^p < 0.01, ^***^p < 0.001.

To further explore the function of CRLF1, we also established a unilateral ureteral obstruction (UUO) model to better simulate the progression of renal fibrosis. The knockdown efficiency of CRLF1 in the kidney was examined (Figure 3A). HE staining revealed severe tubular dilation after the operation. Notably, these pathological changes were alleviated in CRLF1‐knockdown mice (Figure 3B). Masson staining, Sirius Red and IHC staining further demonstrated reduced interstitial fibrosis in CRLF1‐knockdown mice compared to the sham group (Figure 3C–E). qRT‐PCR and Western blotting analysis of *Ngal*, *Vimentin* and *α‐SMA* also showed the expression levels were significantly decreased in CRLF1‐knockdown mice (Figure 3F,G). Immunofluorescence staining of fibrosis indicators Fibronectin and α‐SMA also showed that the expression intensities of these indicators were significantly reduced in CRLF1‐knockdown mice (Figure 3H,I). These findings confirmed the protective role of CRLF1 knockdown against renal fibrosis progression.

### 3.4 Depletion of CRLF1 Attenuates Fibrosis of Cultured Human Tubular Epithelial Cells Following TGF‐β1 Stimulation

**FIGURE 4:**
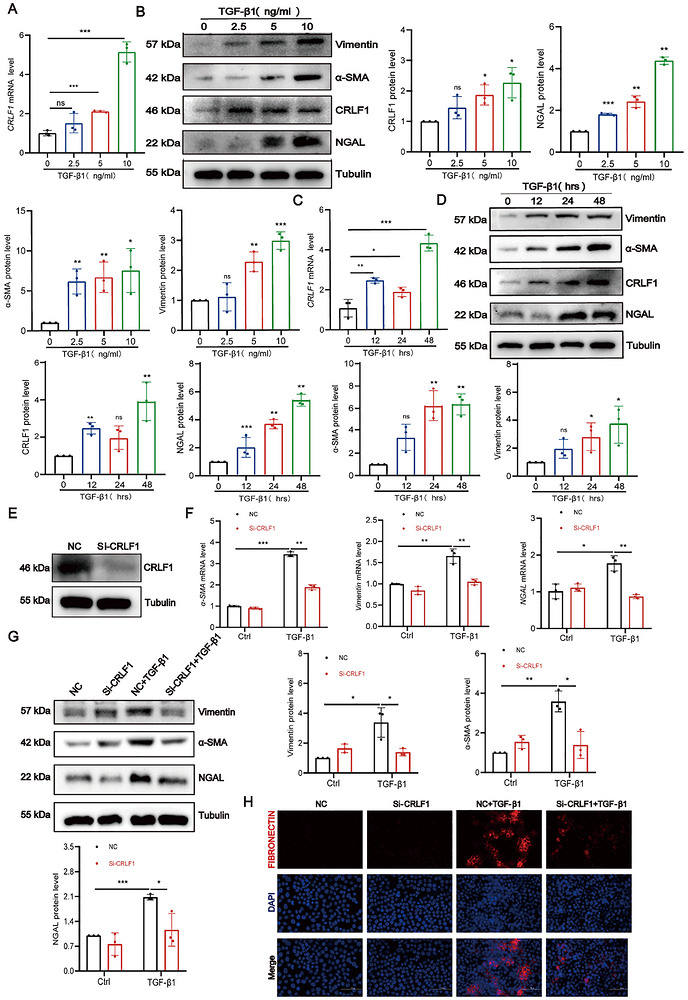
Genetic depletion of CRLF1 inhibits TGF‐β1‐induced fibrosis of HK‐2 cells. (A) qRT‐PCR showed the mRNA expression levels of *CRLF1* in HK‐2 cells stimulated by TGF‐β1 at different concentration gradients. (*n* = 3). (B) Representative Western blotting images and quantification of CRLF1 in HK‐2 cells stimulated by TGF‐β1 at different concentration gradients. (n = 3). (C) qRT‐PCR showed the mRNA expression levels of *CRLF1* in HK‐2 cells stimulated by TGF‐β1 at different time points. (*n* = 3). (D) Representative Western blotting images and quantification of CRLF1 in HK‐2 cells stimulated with TGF‐β1 at different time points. (*n* = 3). (E) Transfection efficiency of NC and Si‐CRLF1 in HK‐2 cells. (F) qRT‐PCR showed the mRNA expression levels of *Vimentin*, *α‐SMA* and *NGAL* in HK‐2 cells transfected with NC or Si‐CRLF1. (*n* = 3). (G) Representative Western blotting images and quantification of Vimentin, α‐SMA and NGAL in HK‐2 cells transfected with NC or Si‐CRLF1. (*n* = 3). (H) Representative IF images showing fibronectin expression levels. Scale bar =  100 µm. Data are shown as the mean ± SD. ^*^p < 0.05, ^**^p < 0.01, ^***^p < 0.001.

In vitro, to explore the function of CRLF1 in AKI‐to‐CKD transition, we utilized small interfering RNA (siRNA) targeting *CRLF1* on TGF‐β1‐induced fibrosis in HK‐2 cells. As shown in Figure 4A,C, TGF‐β1 increased the expression of CRLF1, and Si‐CRLF1 effectively downregulated *CRLF1* mRNA and protein levels (Figure 4E). TGF‐β1 stimulation significantly increased the expression of α‐SMA and Vimentin expression levels (Figure 4B,D), blockade of CRLF1 inhibited upregulation of fibrosis‐related protein (Figure 4F,G). Immunofluorescent staining revealed that the expression of fibronectin was significantly elevated in renal tubular cells under the stimulation of TGF‐β1. However, interference with CRLF1 significantly reduced the expression levels of fibronectin (Figure 4H). Furthermore, flow cytometric analysis quantified the TGF‐β1‐induced G2/M phase arrest, which was significantly reduced by CRLF1 knockdown (Figure S4A). ELISA results showed that the level of CRLF1 in the cell culture supernatant from TGF‐β1‐treated cells was significantly higher than that in the control group (Figure S5A). The combined data indicate that CRLF1 plays a crucial role in the fibrosis in HK‐2 cells.

### 3.5 CRLF1 Activates Renal Interstitial Fibrosis Progression via the PI3K‐AKT Pathway In Vivo and In Vitro

**FIGURE 5:**
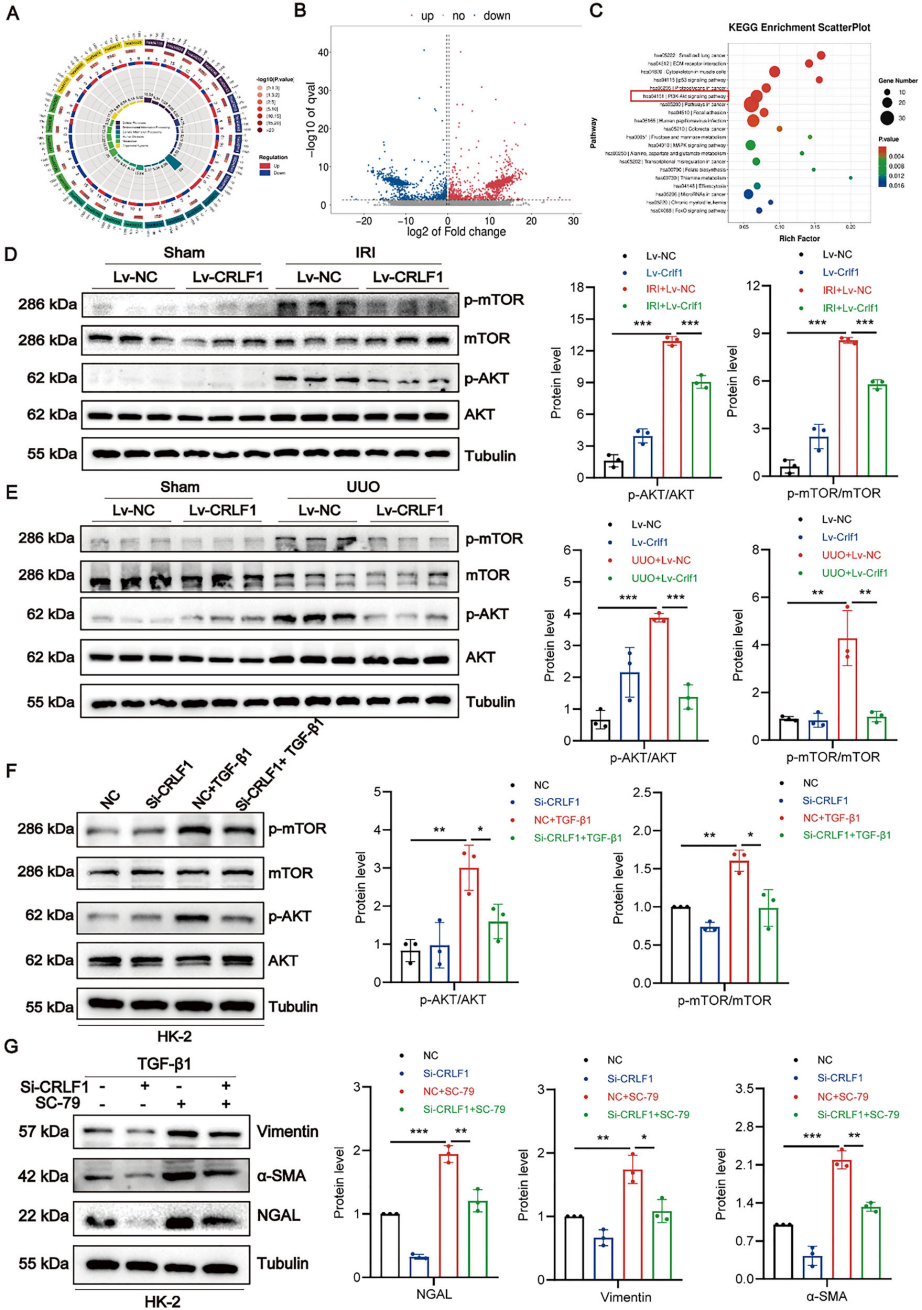
RNA‐seq analysis reveals PI3K‐AKT pathways regulated by CRLF1. (A) Loopcircos plot of DEGs in HK‐2 cells transfected with NC or Si‐CRLF1. (B) Volcano plots showing the DEGs (DEGs: |log2FC|>1, adjusted *p* < 0.05). (C) KEGG enrichment analysis of the PI3K‐AKT signaling pathway. (D) Representative Western blotting images and quantification of p‐AKT (Ser473), AKT, p‐mTOR, mTOR levels in IRI‐induced kidney injury treated as shown. (*n* = 3). (E) Representative Western blotting images and quantification of p‐AKT (Ser473), AKT, p‐mTOR, mTOR levels in UUO‐induced kidney injury treated as shown. (*n* = 3). (F) Representative Western blotting images and quantification of p‐AKT (Ser473), AKT, p‐mTOR, mTOR levels in HK‐2 cells treated as shown. (*n* = 3). (G) Representative Western blotting images and quantification of p‐AKT (Ser473), AKT, p‐mTOR, mTOR levels in HK‐2 cells treated with SC79 as shown. (*n* = 3). Data are presented as the mean ± SD. ^*^p < 0.05, ^**^p < 0.01, ^***^p < 0.001.

To further understand the mechanism through which CRLF1 induces renal fibrosis in renal tubular epithelial cells, we performed RNA sequencing (RNA‐seq) on TGF‐β1‐treated HK‐2 cells transfected with NC or Si‐CRLF1. Gene Ontology (GO) and Kyoto Encyclopedia of Genes and Genomes (KEGG) enrichment analysis of DEGs revealed their main biological functions and biological processes (Figure 5A). Volcano plot analysis results indicate that 406 genes are upregulated and 409 genes are downregulated following CRLF1 silencing (Figure 5B). Subsequently, KEGG enrichment analysis identified the PI3K‐AKT signaling pathway (Figure 5C). Previous studies have reported that activation of the PI3K‐AKT signaling pathway can lead to the progression of AKI to CKD [32, 33, 34]. To validate these conclusions at thetissue level, we detected proteins in mouse renal tissues and observed a significant reduction of p‐AKT (Ser473) and p‐mTOR expression in the IRI‐KD and UUO‐KD groups relative to the Sham group (Figure 5D,E). As illustrated in Figure 5F, the protein levels of p‐AKT (Ser473) and p‐mTOR were decreased in the TGF‐β1‐treated Si‐CRLF1 group compared with the NC group. qRT‐PCR analysis confirmed that CRLF1 knockdown decreased the expression of S6K1, a key gene associated with fibrosis in the PI3K/AKT pathway (Figure S6A). To verify whether CRLF1 mediates TGF‐β1‐induced renal fibrosis via the AKT pathway, HK‐2 cells were treated with TGF‐β1 in the presence or absence of CRLF1 knockdown and/or the AKT activator SC‐79. Silencing of CRLF1 markedly attenuated this profibrotic effect, whereas treatment with SC‐79 reversed this attenuation. (Figure 5G). Treatment of TGF‐β1‐stimulated HK‐2 cells with the AKT inhibitor LY294002 significantly attenuated the upregulation of fibrosis‐related markers and the renal injury marker NGAL (Figure S7A). These results revealed that CRLF1 promotes renal fibrosis via PI3K‐AKT pathway activation in renal tubular epithelial cells.

### 3.6 CRLF1 Directly Binds to ITGB1, and ITGB1 Drives Fibrosis Progression in HK‐2 Cells

**FIGURE 6:**
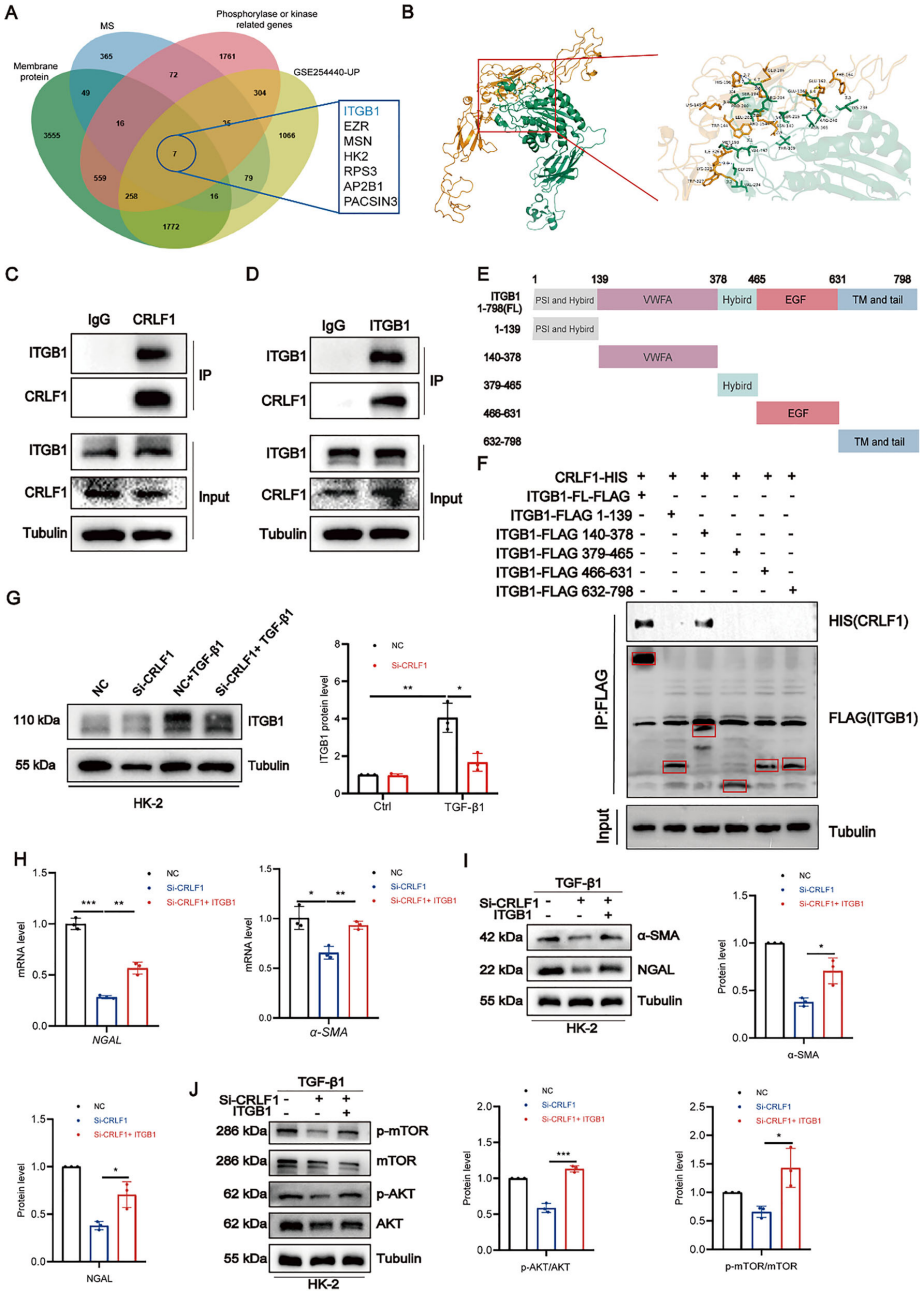
CRLF1 interacts with the VWFA domain of integrin β1. (A) Venn diagram analysis of MS data, Phosphorylase or kinase related genes, genes upregulated in CKD, and membrane proteins. (B) Molecular docking of CRLF1 to ITGB1 predicted with HDOCK. (C) Co‐IP was performed to confirm the binding of CRLF1 to ITGB1. (*n* = 3). (D) Co‐IP was performed to confirm the binding of ITGB1 to CRLF1. (*n* = 3). (E) CRLF1‐His and functional domains of ITGB1‐FLAG expression constructs were co‐transfected into HEK293T cells. (F) Co‐IP was performed to analyze the binding of various ITGB1 truncates (including amino acids 1–139, 140–378, 379–465, 466–631, and 632–798) to full‐length CRLF1 in HEK293T cells. (*n* = 3). (G) Representative Western blotting images and quantification of ITGB1 levels in HK‐2 cells, as shown in the figure. (*n* = 3). (H) qRT‐PCR showed the mRNA expression levels of *NGAL* and *α‐SMA* in HK‐2 cells transfected with or without ITGB1 as indicated. (*n* = 3). (I) Representative Western blotting images and quantification of NGAL and α‐SMA levels in HK‐2 cells, as shown in the figure. (*n* = 3). (J) Representative Western blotting images and quantification of p‐AKT (Ser473), AKT, p‐mTOR and mTOR in HK‐2 cells as indicated. (*n* = 3). Data are presented as the mean ± SD. ^*^p < 0.05, ^**^p < 0.01, ^***^p < 0.001.

Although we have established that CRLF1 can induce renal fibrosis by activating the PI3K‐AKT signaling pathway, the specific mechanisms by which CRLF1 activates this pathway remain unclear. To identify effector proteins of CRLF1, immunoprecipitation (IP) and affinity‐based mass spectrometry (MS) analyses were performed on HK‐2 cells to detect potential CRLF1‐binding partners. We cross‐analyzed mass spectrometry data with genes upregulated in CKD, focusing on membrane proteins and phosphorylation‐related genes. This analysis identified seven proteins: ITGB1, EZR, MSN, HK2, RPS3, AP2B1 and PACSIN3 (Figure 6A). Through HDOCK protein docking, we identified the potential binding sites between ITGB1 and CRLF1 (Figure 6B), while previous studies have reported that ITGB1 is a key protein involved in AKT phosphorylation [35, 36]. We further validated the MS results and demonstrated that CRLF1 protein indeed interacted with ITGB1 (Figure 6C,D). To determine the molecular domains responsible for the CRLF1‐ITGB1 interaction, truncated mutants of ITGB1 were constructed for Co‐IP assays. CRLF1 was co‐transfected with full‐length Flag‐ITGB1, Flag‐ITGB1 (1–139), Flag‐ITGB1 (140–378), Flag‐ITGB1 (379–465), Flag‐ITGB1 (466–631) and Flag‐ITGB1 (632–798) in HEK293T cells (Figure 6E). Co‐IP experiments using anti‐Flag antibody demonstrated that an ITGB1 fragment of ITGB1 (140–378) binds significantly to CRLF1 (Figure 6F). These findings indicate that CRLF1 interacts with ITGB1 primarily through the VWFA (140‐378) domain of ITGB1.

Previous studies have shown that ITGB1 expression in the kidney positively correlates with renal IRI progression [37]. In this study, we observed that inhibition of CRLF1 expression reduced ITGB1 expression under TGF‐β1 stimulation (Figure 6G). To clarify the mechanism, we performed MG132 rescue and ubiquitination assays. The reduction in ITGB1 protein levels caused by CRLF1 knockdown was attenuated by MG132 treatment, accompanied by significantly increased ITGB1 ubiquitination (Figure S8A,B). Thus, CRLF1 stabilizes ITGB1 by inhibiting its ubiquitin‐proteasomal degradation. To determine the function of ITGB1 in vitro, we tested whether oe‐ITGB1 could reverse the renal fibrosis alleviated by Si‐CRLF1. We detected the overexpression effect of ITGB1 (Figure S9A–D). qRT‐PCR and Western blotting results showed that ITGB1 overexpression could significantly increase the expression of NGAL and α‐SMA, which were decreased by Si‐CRLF1 in TGF‐β1‐induced HK‐2 cells (Figure 6H,I). Moreover, ITGB1 overexpression promoted activation of the PI3K‐AKT signaling pathway, the decreased expression of p‐AKT (Ser473) and p‐mTOR caused by Si‐CRLF1 were abolished by ITGB1 (Figure 6J). The expression levels of fibrosis‐related markers α‐SMA and Vimentin, as well as the renal injury marker NGAL, were significantly elevated following stimulation of HK‐2 cells with recombinant CRLF1, and the ITGB1‐AKT pathway was affected and activated (Figure S10A). These results indicate that CRLF1 activates the PI3K‐AKT pathway by binding to the VWFA domain of ITGB1.

### 3.7 SMAD3, as a Transcription Factor (TF), Modulates the Expression of CRLF1

**FIGURE 7:**
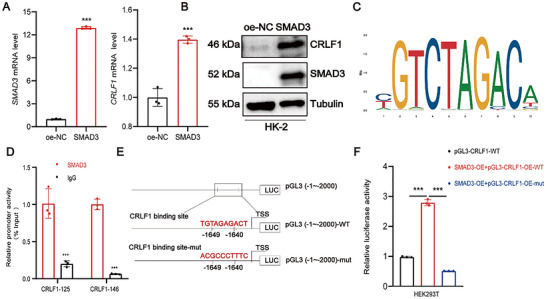
SMAD3 binds to the *CRLF1* promoter and regulates its transcription. (A) qRT‐PCR showed the mRNA expression levels of *CRLF1* in HK‐2 cells transfected with oe‐NC or SMAD3. (*n* = 3). (B) Representative Western blotting images and quantification of CRLF1 in HK‐2 cells transfected with SMAD3‐NC or SMAD3. (*n* = 3). (C) Predicted SMAD3 transcriptional binding sites from the JASPAR database. (D) ChIP‐qPCR assays using SMAD3 antibody were performed to detect SMAD3 binding to the *CRLF1* promoter. Input was used for normalization, and IgG served as a negative control. (*n* = 3). (E) Based on the predicted SMAD3 binding site within the *CRLF1* promoter region, wild‐type (WT) and mutant (MUT) luciferase reporter vectors were constructed. (F) Dual‐luciferase reporter assay results showed the luciferase activity in HEK293T cells co‐transfected with SMAD3 together with wild‐type or mutant CRLF1. (*n* = 3). Data are presented as the mean ± SD. ^*^p < 0.05, ^**^p < 0.01, ^***^p < 0.001.

Previous reports have indicated that SMAD3 can act as a transcriptional regulator of CRLF1 [21]. To elucidate the mechanism by which CRLF1 exacerbates fibrosis, we investigated if SMAD3 could bind to the *CRLF1* promoter. qRT‐PCR indicated an increase in *CRLF1* expression following SMAD3 overexpression in HEK293T cells (Figure 7A). Moreover, Western blotting demonstrated that CRLF1 expression was markedly elevated by SMAD3 overexpression under TGF‐β1 treatment (Figure 7B). Correspondingly, pharmacological inhibition of SMAD3 with SIS3 markedly reduced CRLF1 protein and mRNA levels in HK‐2 cells (Figure S11A,B). These findings indicate that SMAD3 can function as a transcriptional regulator of CRLF1 to promote its transcription. Analysis using the JASPAR database (https://jaspar.genereg.net/) identified two potential SMAD3 binding motifs within the promoter region of the *CRLF1* gene (Figure 7C). We performed ChIP with the anti‐SMAD3 antibody on HK‐2 cell extracts, followed by ChIP‐qPCR on the pulled‐down DNA with specific primers designed to amplify these predicted sites in the promoter region of *CRLF1*. Primer set 125 and Primer set 146 generated a PCR product from the ChIP isolated DNA, indicating that these regions directly bind to SMAD3 (Figure 7D). Given the greater impact of primer set 146, we selected this sequence for next exploration. Subsequently, we inserted wild‐type and mutated primer set 146 sequences into a dual luciferase reporter construct and evaluated their transcriptional activity in a functional assay. Transfection of these constructs into HEK293T cells resulted in wild‐type sequence‐induced transcription, whereas the mutant binding site exhibited no such effect (Figure 7E,F). Collectively, these results demonstrate that SMAD3 acts as a transcription factor for CRLF1 and promotes its transcription.

## 4 Discussion

AKI is a prevalent and high‐risk syndrome with high incidence and mortality, often leading to renal fibrosis and progression to CKD [38, 39, 40]. The precise mechanisms that lead to damage of renal TECs and trigger tubulointerstitial fibrosis during the progression from AKI to CKD remain incompletely elucidated. CRLF1 expression is elevated in renal tubular epithelial cells of UUO and IRI conditions, and its expression plays a crucial regulatory role in the progression of tubulointerstitial fibrosis. Mechanistically, we found that SMAD3 can bind to the promoter of CRLF1and promote its transcription, thereby upregulating the expression of fibrosis‐related genes. As a secreted protein, CRLF1 primarily promotes the progression of renal fibrosis by interacting with the VWFA domain (140‐378) of ITGB1. This mechanism seems to be pivotal in the AKI‐to‐CKD transition.

As a secreted protein, CRLF1 forms heterodimers with cardiotrophin‐like cytokine factor 1 (CLCF1) [16]. These dimers bind to the ciliary neurotrophic factor receptor (CNTFR), thereby completing cellular signal transduction. However, some researchers have questioned CRLF1's role as a ligand and suggested that CRLF1 may possess functions beyond serving as a ligand for CLCF1 [17]. The expression level of CRLF1 positively correlates with the degree of liver fibrosis. Overexpression of CRLF1 significantly upregulates the expression levels of α‐SMA and COL3 in quiescent hepatic stellate cells [18]. CRLF1 is upregulated in myocardial fibrosis and exacerbates myocardial fibrosis by increasing p‐ERK1/2 protein levels [19]. However, the expression and function of CRLF1 in the progression of renal fibrosis remain unclear.

Previous studies have demonstrated that CRLF1 plays a crucial role in inducing fibrosis and inflammation [21, 41, 42]. In this study, we utilized the GEO database to identify CRLF1 as one of the upregulated genes in kidney tissues from AKI and CKD patients, further demonstrating its increased expression in human and mouse AKI‐to‐CKD models. Knockdown of CRLF1 reduced the expression of renal injury and fibrosis‐related markers, indicating that CRLF1 promotes renal fibrosis progression.

The PI3K/AKT signaling pathway is widely activated in tissue cells and plays a crucial role in cell proliferation, differentiation, invasion, migration, and EMT [43]. This pathway exerts significant effects on renal injury by regulating fibroblast differentiation and ECM deposition during fibrosis [44, 45, 46, 47], and is considered one of the most important signaling pathways in the progression of renal fibrosis. The transmembrane glycoprotein ITGB1 is a subunit of the integrin protein family involved in numerous biological processes, including adhesion, migration, cell cycle progression, differentiation, and muscle regeneration [48, 49, 50, 51]. Previous studies have demonstrated that ITGB1 drives the PI3K/AKT pathway, thereby enhancing pancreatic cancer resistance to cetuximab treatment [52]. Our study demonstrates that CRLF1 interacts with ITGB1 to regulate renal fibrosis via the PI3K/AKT signaling pathway, as determined by MS, HDOCK protein docking, and RNA‐seq analyses.

During ligamentum flavum hypertrophy, activation of the TGF‐β1 signaling pathway not only induces collagen production and myofibroblast transformation but also increases CRLF1 expression [21]. SMAD3 promotes CRLF1 expression and thereby drives cardiac fibrosis [19]. The transcription factor SMAD3 also plays a crucial role in renal fibrosis progression. However, the interaction between SMAD3 and CRLF1, as well as their binding characteristics, remains unclear. In our research, ChIP assays confirmed that SMAD3 binds to the CRLF1 promoter, acting as a transcription factor to enhance its transcription. A dual‐luciferase reporter assay further validated the binding site.

In the present study, we demonstrated for the first time the potential mechanism of CRLF1 in AKI‐to‐CKD. During renal fibrosis, SMAD3 acts as a transcription factor for CRLF1, promoting its expression and secretion. CRLF1 is secreted and then binds to ITGB1, activating the PI3K‐AKT signaling pathway to mediate renal fibrosis. This accelerates the progression from AKI to CKD, highlighting the therapeutic potential of targeting CRLF1 to alleviate renal fibrosis. Future research should directly block the interaction between CRLF1 and ITGB1 in vivo as an adjuvant therapy to delay the progression of AKI to CKD. However, the limitation of this study is that, although it provides preliminary evidence that CRLF1 in renal tubular epithelial cells contributes to the progression of renal fibrosis, further validation using renal tubular‐specific CRLF1 knockout mice is needed to better understand its regulatory function in kidney fibrogenesis. In addition, renal fibroblasts exert important and complex effects during renal fibrosis. The specific role of CRLF1 in this process remains to be further investigated in future studies.

## Author Contributions

C.J.W. and Y.Z. contributed equally to this work. C.J.W. and Y.Z. contributed to experimental design, validation, data acquisition, and manuscript writing; F.B. and S.K.Q. contributed to experimental design, validation, and data acquisition; F.F. contributed to data acquisition; F.Y.L., J.H.F. and K.P.Y. contributed to study conception, data acquisition, and manuscript writing; X.D.Y. contributed to manuscript guidance, data interpretation, and supervision. All authors were involved in writing and revising the manuscript and approved the final version for publication.

## Conflicts of Interest

The authors declare no conflicts of interest.

## Supporting information




**Supporting File 1**: advs76896‐sup‐0001‐SuppMat.docx.

**Supporting File 2**: advs76896‐sup‐0002‐TableS1‐S3.docx.

## Data Availability

The data that support the findings of this study are available from the corresponding author upon reasonable request.
