# Supplementary material for: Renal Tubular Epithelial CRLF1 Interacts With ITGB1 to Accelerate Fibrosis During the Transition From AKI to CKD
Source: Adv Sci (Weinh). 2026 Jul 30;13(59):e76896. doi: 10.1002/advs.76896 (PMC13423483; doi:10.1002/advs.76896)
Supplement: Supplementary file 1 [file ADVS-13-e76896-s001.docx]

**Supplementary Table**

**Table S1**

**siRNA/shRNA sequence used in this study**

| **Terms** | **5'-3'** | **3'-5'** |
| --- | --- | --- |
| CRLF1-human-1181 siRNA | GGAUGCAGAAGUCGCACAATT | UUGUGCGACUUCUGCAUCCTT |
| CRLF1-human-428 siRNA | GCUGGUCCAAGAACAUGAATT | UUCAUGUUCUUGGACCAGCTT |
| CRLF1-human-218 siRNA | GCCUCUACUGGACCCUCAATT | UUGAGGGUCCAGUAGAGGCTT |
| Negative control | UUCUCCGAACGUGUCACGUTT | ACGUGACACGUUCGGAGAATT |
| lv-NC-mus | TTCTCCGAACGTGTCACGT |  |
| lv-CRLF1-mus | GGTGGTACGGTCAGGATAA |  |

**Table S2**

**experimental antibodies**

| **Antibodies** | **SOURCE** | **IDENTIFIER** |
| --- | --- | --- |
| mTOR | CST | 2983S |
| Phosphorylation mTOR | CST | 5536S |
| α-SMA | Abcam | ab124964 |
| NGAL | Abcam | ab318209 |
| AQP1 | Santa Claus | Sc-25287 |
| Phosphorylation SMAD3 | CST | 9520S |
| HIS | CST | 2366T |
| AKT | CST | 9272S |
| Phosphorylation AKT(S473) | CST | 4060T |
| CRLF1 | Abcam | ab211438 |
| CRLF1 | Abmart | **PC8663** |
| DYKDDDDK | Proteintech | 20543-1-AP |
| Alexa Fluor594 Goat anti Rabbit IgG | Proteintech | SA00006-4 |
| AlexaFluor488 Goat anti Rabbit IgG | Proteintech | SA00006-2 |
| ITGB1 | Proteintech | 12594-1-AP |
| Tubulin | Proteintech | 66031-1-lg |
| VIMENTIN | Proteintech | 10366-1-AP |
| Goat anti-rabbit IgG-HRP | Proteintech | SA00001-2 |
| Goat anti-mouse IgG-HRP | Proteintech | SA00001-1 |

**Table S3**

**Primers and RNA sequences used in this study.**

| **Terms** | **Forward primer (5'-3')** | **Reverse primer (3'-5')** |
| --- | --- | --- |
| Mouse Crlf1 | GGCACCGTTTACTTCGTCCA | GCTCCAGATTCCCGCCTTT |
| Mouse IL-1β | GCTGAGGAAGATGCTGGTTC | TCCATATCCTGTCCCTGGAG |
| Mouse TNF-α | ATCAGAGGGCCTGTACCTCA | GGAAGACCCCTCCCAGATAG |
| Mouse Tubulin | GTACCTACCACGGTGACAGC | CCCAGACTGACCGAAAACGA |
| Mouse VIMENTIN | TCCAGAGAGAGGAAGCCGAA | TTCAAGGTCAAGACGTGCCA |
| Human CRLF1 | CGATGTGAGCAACCAGACCT | GCCATAGATGCCAAAGGGGT |
| Human IL-1β | GCTGAGGAAGATGCTGGTTC | TCCATATCCTGTCCCTGGAG |
| Human TNF-α | GAGGCCAAGCCCTGGTATG | CGGGCCGATTGATCTCAGC |
| Human TUBULIN | CATGGACTCTGTTCGCTCAGG | CCTTTGGCCCAGTTGTTACCT |
| Human α-SMA | TGAGCGTGGCTATTCCTTCG | AGCGTTCGTTTCCAATGGTG |
| Human VIMENTIN | AGGCGAGGAGAGCAGGATTT | AGTGGGTATCAACCAGAGGGA |
| Human SMAD3 | CACGGAGACACATCGGAAGA | CGCTGGTTACAGTTGGGAGA |
| Human ITGB1 | CCAACCTGATCCTGTGTCCC | ACCATGACCTCGTTGTTCCC |
