# Supplementary material for: Renal Tubular Epithelial CRLF1 Interacts With ITGB1 to Accelerate Fibrosis During the Transition From AKI to CKD
Source: Adv Sci (Weinh). 2026 Jul 30:e76896. Online ahead of print. doi: 10.1002/advs.76896 (PMC13423483; doi:10.1002/advs.76896)
Supplement: Supplementary file 1 — Supporting File 1: advs76896‐sup‐0001‐SuppMat.docx. [file ADVS-9999-e76896-s001.docx]

**Supplementary figures and figure legends**


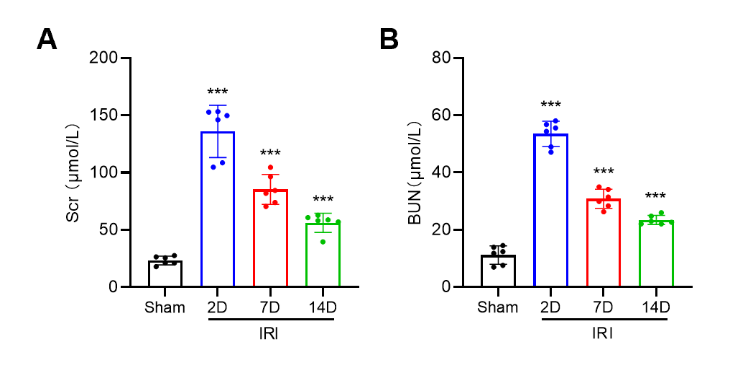


Fig S1: Renal function parameters in mice in the IRI model. (A) Scr concentrations on 2D,7D,14D after IRI. (B) BUN levels on 2D,7D,14D after IRI (n=6). Data are presented as the mean ± SD. ****P < 0.001*.


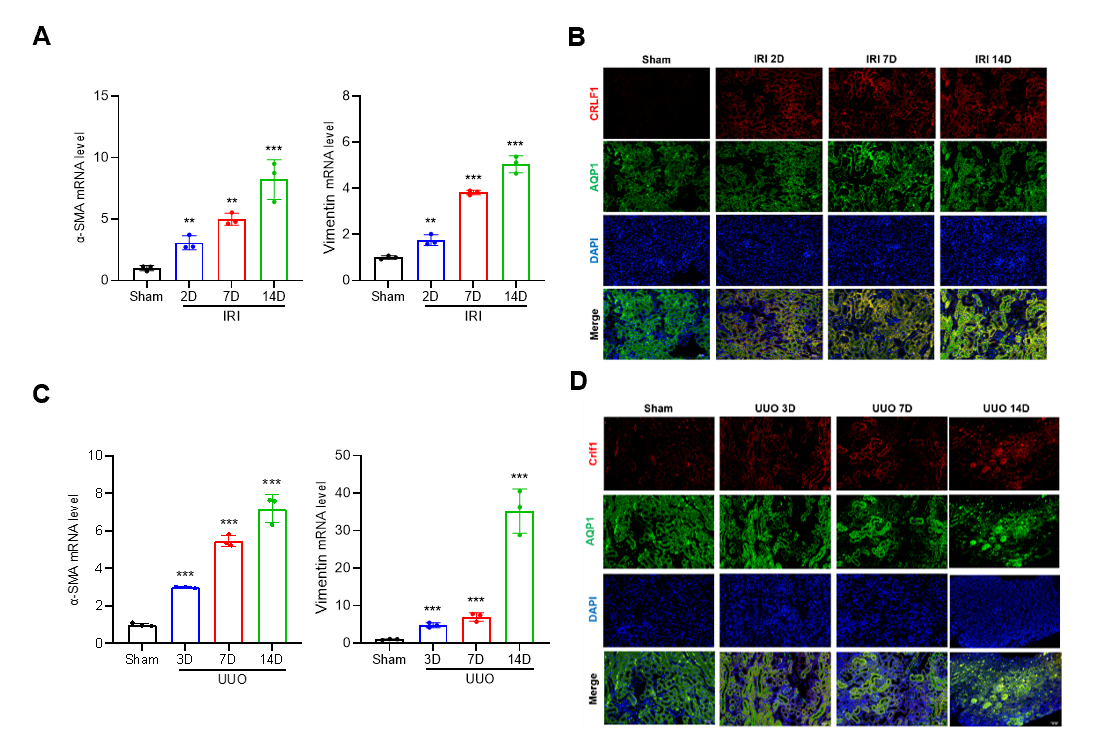


Fig S2: CRLF1 is elevated in IRI and UUO mouse kidneys and colocalizes with AQP1. (A) qRT-PCR showed the mRNA expression levels of α-SMA and Vimentin of kidney tissues on days 0, 2, 7 and 14 in IRI induced AKI-CKD transition mouse model. (n=3). (B) Representative images of IF for CRLF1 and AQP1, which stained the nucleus with DAPI. Scale bar = 50 μm. (C) qRT-PCR showed the mRNA expression levels of α-SMA and Vimentin of kidney tissues on days 0, 3, 7 and 14 in UUO induced mouse model. (n=3). (D) Representative images of IF for CRLF1 and AQP1，which stained the nucleus with DAPI. Scale bar = 50 μm. Data are presented as the mean ± SD. ***P < 0.01, ***P < 0.001*.


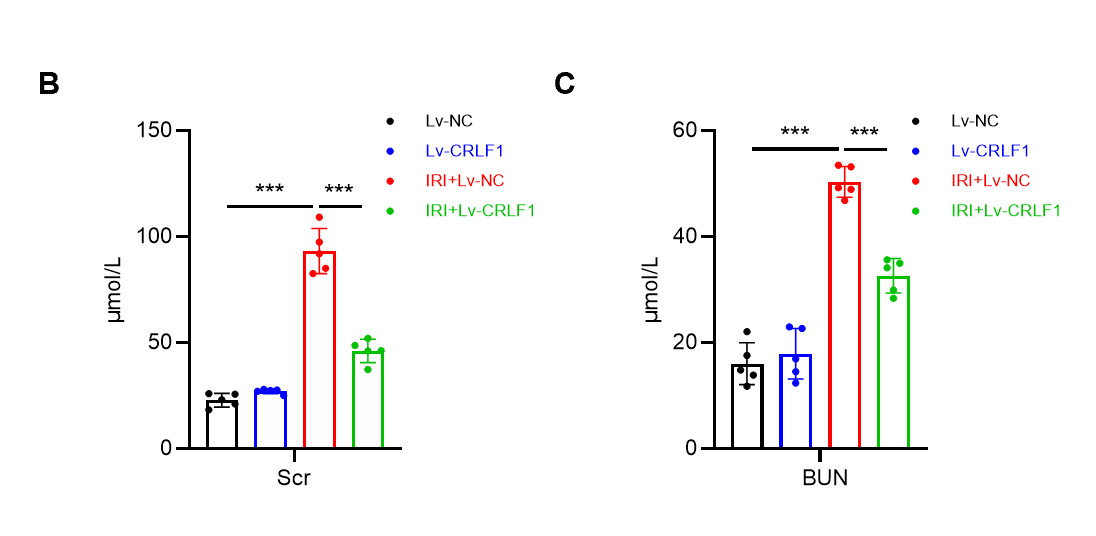

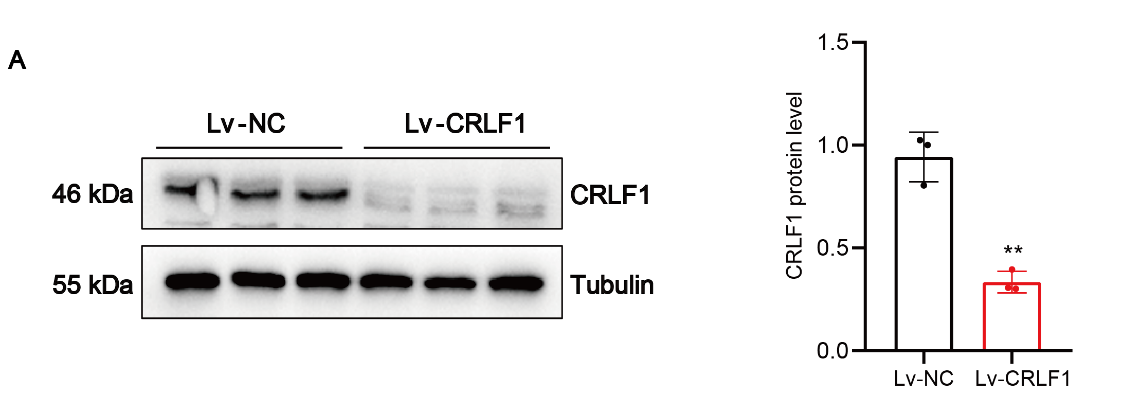


Fig S3: CRLF1 was inhibited by Lentivirus. (A) Representative Western blotting images and quantification of CRLF1 in the two groups of kidneys. (B) Scr concentrations of healthy mice and mice subjected to IRI with or without lv-NC or lv-CRLF1. (C) BUN levels of healthy mice and mice subjected to IRI with or without lv-NC or lv-CRLF1. Data are presented as the mean ± SD. ***P < 0.01*.


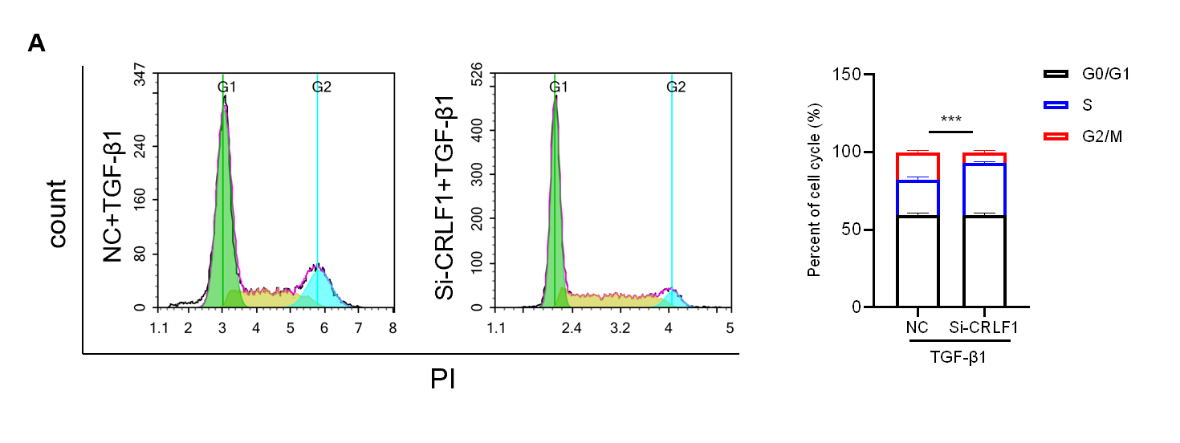


Fig S4: CRLF1 knockdown reduces G2/M phase proportion under TGF-β1 stimulation. (A) Flow cytometric analysis of cell cycle distribution in TGF‑β1‑treated HK-2 cells among different experimental groups (n = 3). Data are presented as the mean ± SD. ****P < 0.001*.


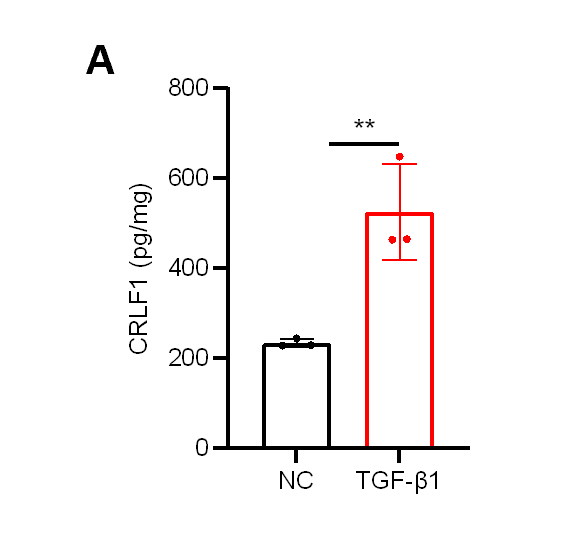


Fig S5: The expression level of CRLF1 was significantly elevated in the supernatant of HK-2 cells. (A) ELISA results showed that CRLF1 expression was significantly upregulated in the culture supernatant of TGF-β1-stimulated HK-2 cells. (n=3). Data are presented as the mean ± SD. ***P < 0.01*.


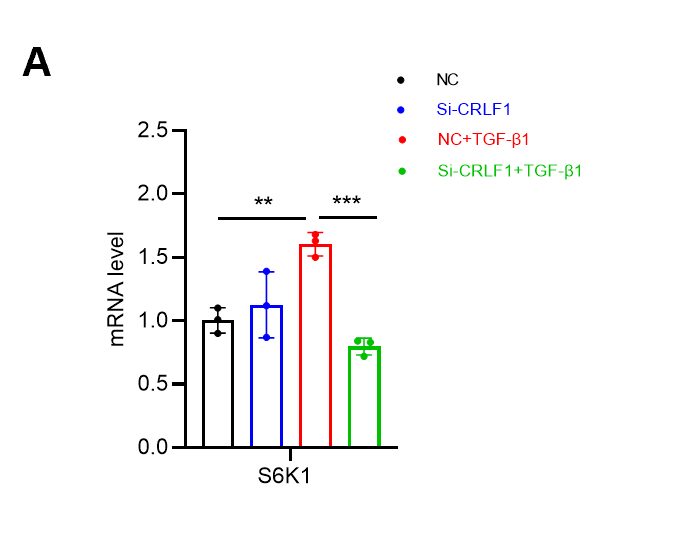


Fig S6: CRLF1 promotes the progression of renal fibrosis via the PI3K-AKT pathway (A) qRT-PCR analysis confirmed that CRLF1 knockdown decreased the expression of S6K1. (n=3). Data are presented as the mean ± SD. ***P < 0.01, ***P < 0.001*.


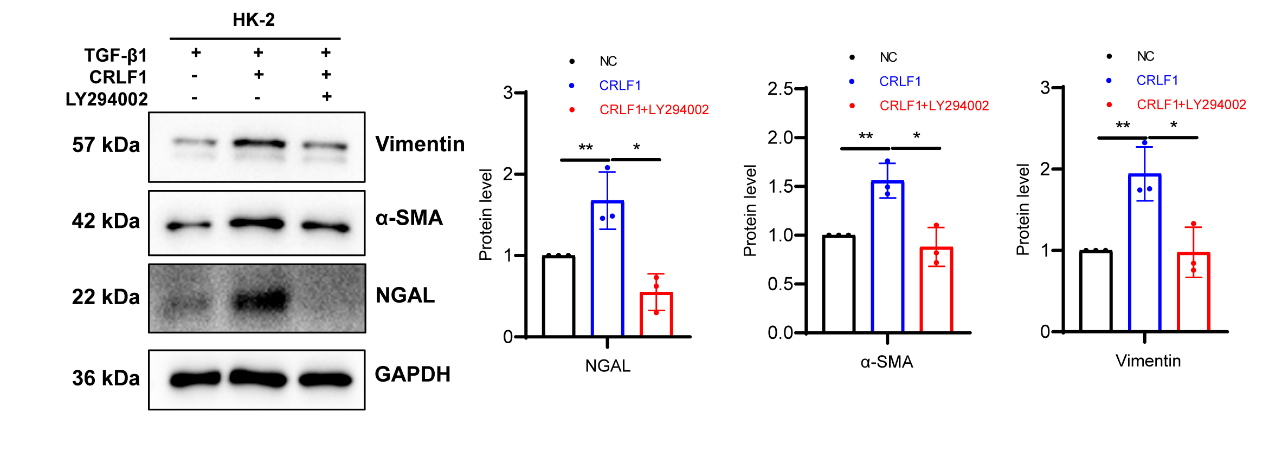


Fig S7: Inhibition of AKT attenuates TGF-β1-induced fibrotic responses in HK-2 cells. (A) Representative Western blotting images and quantification of fibrosis-related markers in HK-2 cells treated with TGF-β1 alone or in combination with the LY294002. Data are presented as the mean ± SD. (n=3). **P < 0.05, **P < 0.01*.


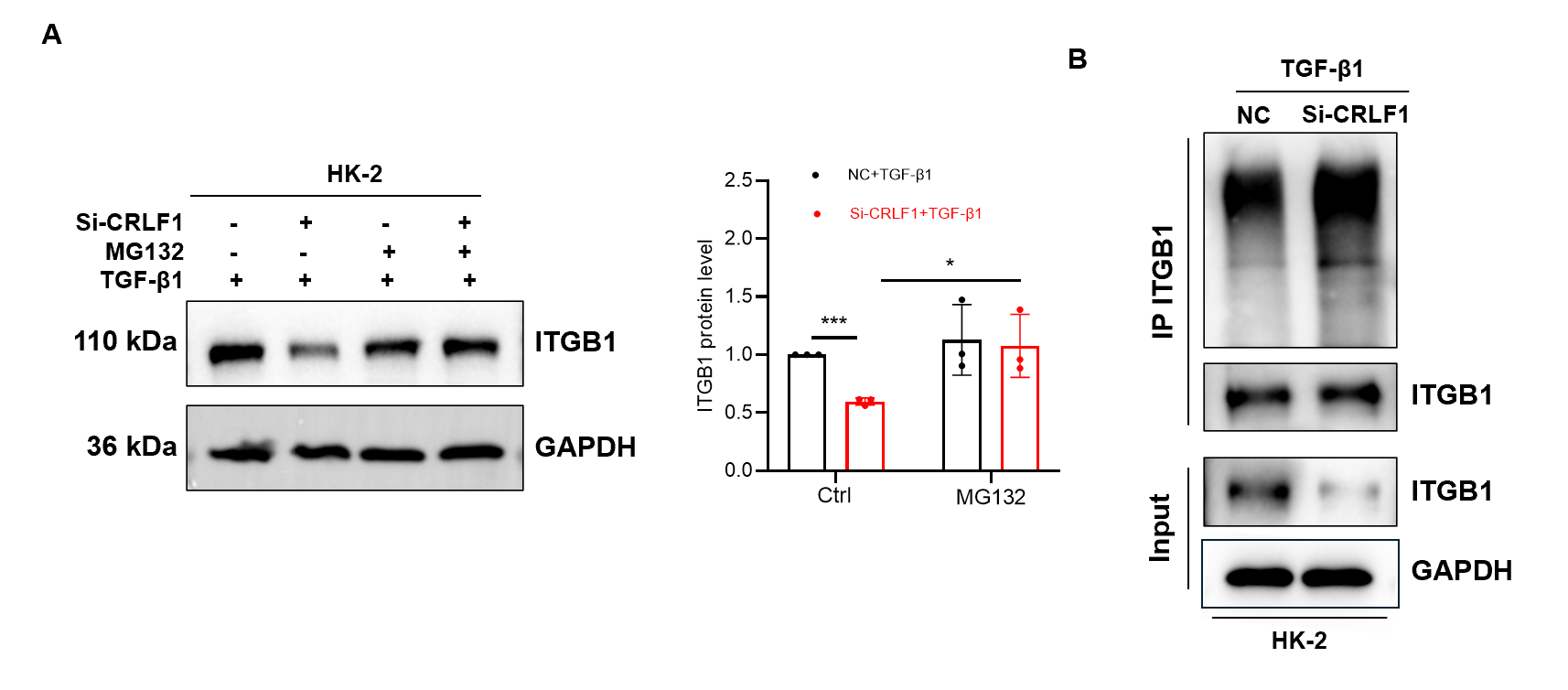


Fig S8: CRLF1 knockdown promotes ITGB1 ubiquitination and proteasomal degradation under TGF-β1 stimulation. (A) Western blotting and quantification showing that CRLF1 silencing reduced ITGB1 protein levels, which was rescued by the proteasome inhibitor MG132. (B) Co‐immunoprecipitation (Co‐IP) experiments showing increased ITGB1 ubiquitination in TGF-β1-treated HK-2 cells upon CRLF1 knockdown. Data are presented as the mean ± SD. **P < 0.05, ***P < 0.001*.


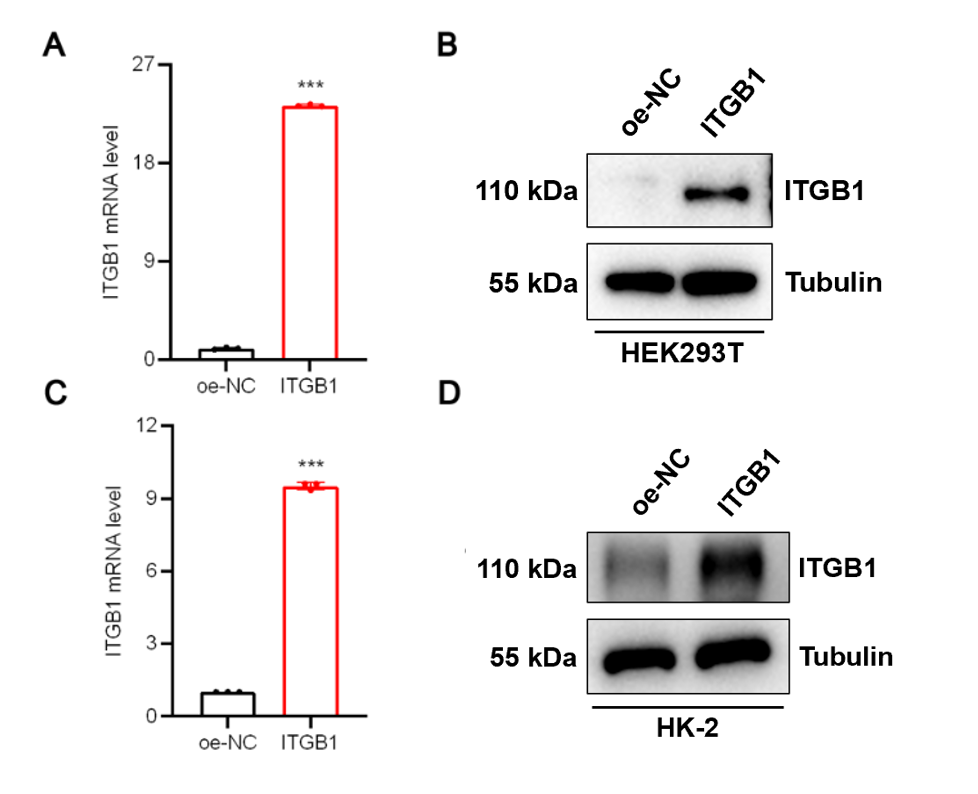


Fig S9: ITGB1 was significantly elevated in HK-2 and HEK293T cells transfected with oe-NC and oe-ITGB1. (A) qRT-PCR showed the mRNA expression levels of ITGB1 in HK-2 cells transfected with oe-NC and oe-ITGB1. (n=3). (B) Representative Western blotting images and quantification of ITGB1 in HK-2 cells transfected with oe-NC and oe-ITGB1. (C) qRT-PCR showed the mRNA expression levels of ITGB1 in HEK293T cells transfected with oe-NC and oe-ITGB1. (n=3). (D) Representative Western blotting images and quantification of ITGB1 in HEK293T cells transfected with oe-NC and oe-ITGB1. (n=3). Data are presented as the mean ± SD. ****P* < 0.001.


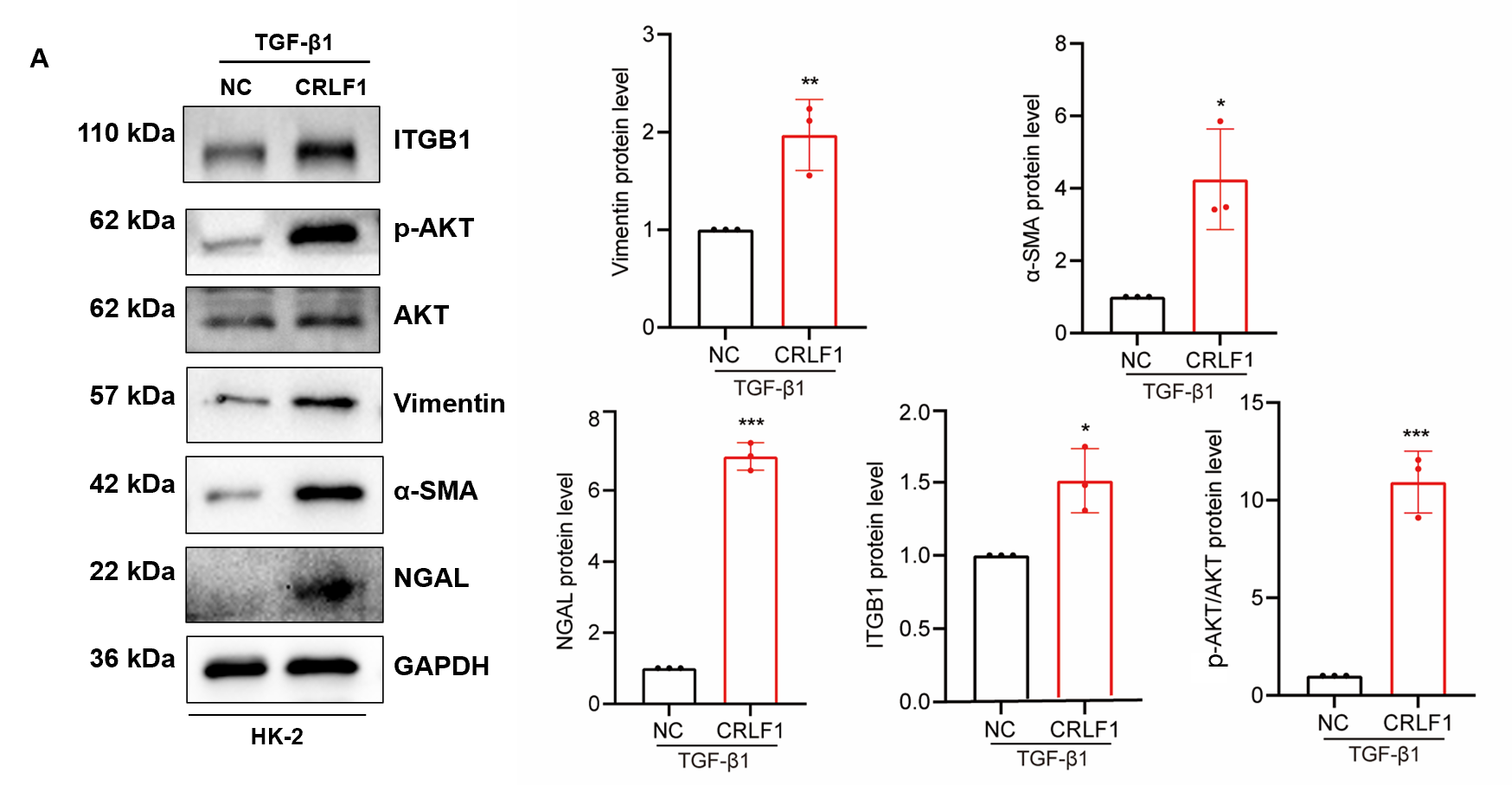


Fig S10: The recombinant CRLF1 protein promotes fibrosis in HK-2 cells and activates the ITGB1–PI3K/AKT pathway. (A) Recombinant CRLF1 protein significantly increased the expression levels of the fibrosis-associated proteins α-SMA and Vimentin following stimulation of HK-2 cells, and the ITGB1–AKT pathway was activated. (n=3). Data are presented as the mean ± SD. **P < 0.05, **P < 0.01, ***P < 0.001.*


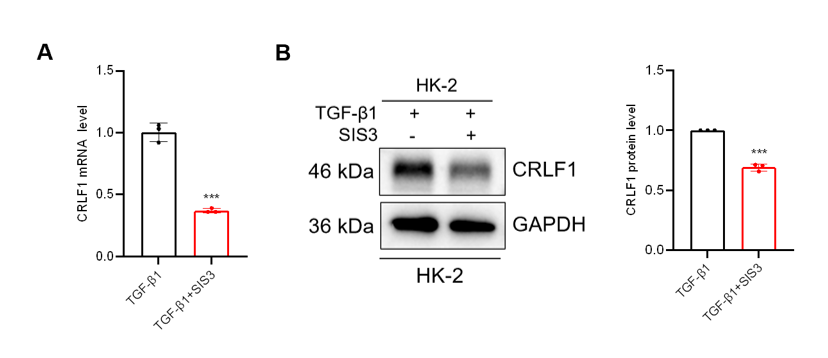


Fig S11: Pharmacological SMAD3 inhibition suppresses CRLF1 upregulation in vitro. (A) qRT-PCR analysis confirmed that SIS3 decreased the expression of CRLF1. (n=3). (B) Representative Western blotting images and quantification of CRLF1 in HK-2 cells treated with SIS3. Data are presented as the mean ± SD. ***P < 0.01, ***P < 0.001*.
